# Inkjet printing infiltration of Ni-Gd:CeO_2_ anodes for low temperature solid oxide fuel cells

**DOI:** 10.1007/s10800-017-1114-x

**Published:** 2017-08-17

**Authors:** C. Wang, R. I. Tomov, T. B. Mitchell-Williams, R. V. Kumar, B. A. Glowacki

**Affiliations:** 1 Department of Materials Science and Metallurgy, University of Cambridge, 27 Charles Babbage Road, Cambridge, CB3 0FS UK; 2 Institute of Power Engineering, Warsaw, Poland

**Keywords:** Solid oxide fuel cells, Infiltration, Inkjet printing, Gadolinium doped ceria, Nanostructure

## Abstract

**Abstract:**

The effect of inkjet printing infiltration of Gd_0.1_Ce_0.9_O_2−*x*_ in NiO-Gd_0.1_Ce_0.9_O_2−*x*_ anodes on the performance of symmetrical and button cells was investigated. The anodes were fabricated by inkjet printing of suspension and sol inks. Symmetrical cells were produced from composite suspension inks on Gd_0.1_Ce_0.9_O_2−*x*_ electrolyte. As-prepared scaffolds were infiltrated with Gd_0.1_Ce_0.9_O_2_ ink. Increasing the number of infiltration steps led to formation of “nano-decoration” on pre-sintered anodes. High resolution SEM analysis was employed for micro-structural characterization revealing formation of fine anode sub-structure with nanoparticle size varying in the range of 50–200 nm. EIS tests were conducted on symmetrical cells in 4% hydrogen/argon gas flow. The measurements showed substantial reduction of the activation polarization as a function of the number of infiltrations. The effect was assigned to the extension of the triple phase boundary. The *i*–*V* testing of a reference (NiO-8 mol% Y_2_O_3_ stabilized ZrO_2_/NiO-Gd_0.1_Ce_0.9_O_2−*x*_/Gd_0.1_Ce_0.9_O_2−*x*_/Gd_0.1_Ce_0.9_O_2−*x*_-La_0.6_Sr_0.4_Co_0.2_Fe_0.8_O_3−*δ*_) cell and an identical cell with infiltrated anode revealed ~2.5 times improvement in the maximum output power at 600 °C which corresponded with the reduction of the polarization resistance of the symmetrical cells at the same temperature (2.8 times). This study demonstrated the potential of inkjet printing technology as an infiltration tool for cost effective commercial SOFC processing.

**Graphical abstract:**

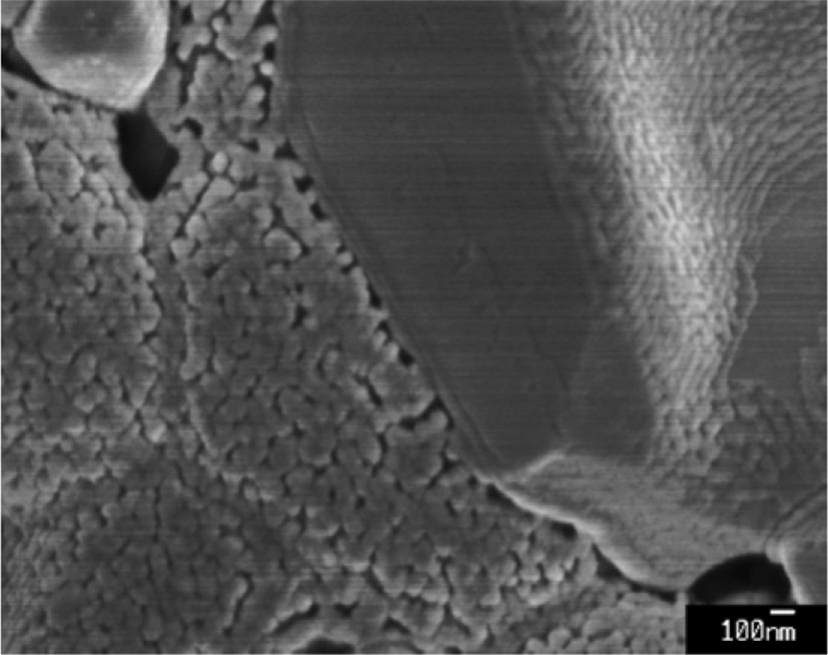

## Introduction

Solid oxide fuel cells (SOFCs) have been an object of continuous research efforts for decades due to their highly efficient direct conversion of chemical energy into electricity, fuel flexibility and environmental benefits. SOFCs cover a wide range of applications from auxiliary power units (1–10 kW) and combined heat and power plants (hundreds of kW) to stationary installations (MW range) [1]. Depending on the design, SOFCs can operate at various temperatures within the region of 500–1000 °C [2, 3]. Anode-supported SOFCs are considered the best choice for operation at reduced temperature having substantially thinner electrolytes and hence much lower ohmic resistance compared to the electrolyte supported cells. The state-of-the-art commercial anode-supported SOFCs are based on a combination of ion-conducting ceramic electrolyte materials (doped ZrO_2_, doped CeO_2_) and cermet anodes (e.g. Ni-doped ZrO_2_, Ni-doped CeO_2_). Both electrolyte materials offer good chemical and thermal stability in oxidizing and reducing atmospheres and high oxygen ionic conductivity over a wide range of conditions [4, 5]. Ni based cermet anodes are preferred due to their good combination of sufficient electrical conductivity and mechanical strength, as well as minimal chemical interaction with the electrolyte [6, 7]. Commercial anode-supported SOFCs operate at levels of 800–1000 °C which introduces severe limitations in the choice of materials and manufacturing technologies. Such high operating temperatures require utilization of expensive corrosion resistant interconnects and are detrimental to the durability of the cells. As a consequence, the main barriers for SOFC commercialization are the high production costs and limited operational lifetimes. Hence, using lower operational temperatures (<800 °C) is considered essential for the extensive commercialisation of SOFC technology. The advantages of reduced-temperature operation include better system design compactness as well as wider choice of materials [7, 8]. It permits utilization of less expensive stainless steel (as support and interconnector materials) and more reliable sealing solutions. Various strategies have been developed for compensation of the drop in ionic conductivity incurred by the reduced operational temperature. These approaches concentrate on lowering the electrolyte resistance either by implementing thinner layers or using materials with higher ionic conductivity, as well as reduction of the activation polarization losses and enhancement of the electrochemical activity of the electrodes [7, 8]. Doped ceria has been proposed as an electrolyte of choice for lower operating temperatures (550–650 °C) [9, 10] as it offers ionic conductivities significantly higher than those of 8 mol% Y_2_O_3_ stabilized ZrO_2_ (8YSZ) at 600 °C. Adversely, the use of doped ceria as electrolyte is constrained for operating temperatures below 650 °C as it exhibits substantial electronic conduction at higher temperatures [11]. Lower operating temperatures have the advantage of inhibiting detrimental electrode degradation. However, they lead to an exponential decrease of the thermally activated electrodes’ reaction rates as the polarization losses at the anode and the cathode becomes critical. The enhancement of the electrochemical activity of both electrodes via infiltration with active precursor inks is considered to be a promising route to commercialization of low and intermediate temperatures SOFCs.

In the last decade, a number of studies have been focused on micro-structural optimization of SOFC electrodes via infiltration of catalytic and non-catalytic precursors into the porous electrode scaffolds. Several detailed review papers have been published summarising some exceptionally encouraging results [6, 8, 12–18]. The infiltrated inks were usually calcined at relatively low temperature, typically below 800 °C, resulting in formation of distributed nanoparticles without detrimental chemical reaction between the scaffold and the infiltrate material. Such “nano-decoration” effectively extended the triple phase boundary (TPB) density minimizing the polarization losses of the electrodes. The effort directed towards finding effective cathode nano-engineering solutions has been motivated by the higher activation energy of the cathodic oxygen reduction reaction (ORR). However, at low operating temperatures polarization losses in the cermet anodes become significant. Such anodes are also vulnerable to degradation during SOFC operation, as the metal catalytic particles (typically Ni) tend to coarsen by sintering, effectively reducing the length of the TPB. Thus an extension of the anode TPB length and an improvement of the anode performance stability have been pursued by infiltration of ionic conductive phases, e.g. Gd_0.1_Ce_0.9_O_2−*x*_ (GDC) and/or catalytic metal phases (e.g. Ni) into the anode scaffolds. The ionic conductive material infiltration tended to produce better long-term enhancement of the anodes’ electrochemical activity due to the lower degree of nanoparticles coarsening [12, 17, 19]. It was also reported that the infiltration of ceria based inks inhibited the grain growth of the original constituent particles of the anode scaffolds thus improving the anodes’ long-term performances [14].

Jiang et al. infiltrated pure Ni anodes with YSZ and doped-CeO_2_ investigating the effect of the resulting nano-decoration on the electrode microstructure and the cell performances [20–22].The electro-catalytic activity of the infiltrated anodes in the hydrogen oxidation reaction was found to be substantially enhanced, i.e. after impregnation with 8.5 vol% GDC (1.7 mg cm^−2^), the electrode polarization resistance of pure Ni anodes was reduced ~7 times to *R*_p_ = 1.5 Ω cm^2^ at 700 °C and to *R*_p_ = 0.71 Ω cm^2^ at 800 °C. In an earlier report, a triple vacuum infiltration of composite (Ni-TZ8Y cermet) anodes with 3 M Sm_0.2_Ce_0.8_(NO_3_)_*x*_ solution successfully reduced the anode polarization resistance to *R*_p_ = 0.24 Ω cm^2^ at 800 °C [23], effectively lowering the operating temperature of the anodes by 200 °C compared to the anodes without an impregnation treatment. It was suggested that the infiltration of oxide particles such as YSZ and doped ceria into the cermet structure extended the TPB areas thus significantly enhancing the electrode performance. Liu et al. [24] infiltrated water based inks of Sm_2_O_3_, Sm_0.2_Ce_0.8_O_*x*_ (SDC), and non-doped ceria (CeO_2_) into bi-layer pellets consisting of porous NiO-SDC substrates and dense SDC electrolytes. They found that for an optimum loading level of 1.71 mmol cm^−3^ SDC ink and after four cycles of impregnation/heating treatment, the peak power density of the button cells increased ~1.3 times to 754 mW cm^−2^ at 800 °C and the combined polarization resistance decreased ~1.3 times to *R*_p_ = 0.216 Ω cm^2^ . Based on infiltration data for all other infiltrated oxides the authors concluded that the improved performances could be mainly attributed to the significantly enhanced catalytic activities of the anodes, but not to the extension of TPB. Timurkutluk et al. [25] infiltrated GDC ink into the anodes and the cathodes of NiO-Sc stabilized ZrO_2_(ScSZ)/ScSZ/La_0.60_Sr_0.40_FeO_3−*d*_(LSF)-ScSZ button cells. After multiple infiltrations, it was found that at 700 °C, the cell impregnated with 1.5 M solution provided peak power density of ~1.34 W cm^−2^ compared to the cell without impregnation producing only 0.78 W cm^−2^ peak power density. As reported data suggested that the observed performance improvements could be attributed to both contributions discussed above—extended TPB and enhanced catalytic activity. Lee et al. [26] demonstrated a method for creation of bi-modally integrated anode functional layer by infiltrating a very small amount (~2 wt%) of Ni and GDC mixed precursor solution into a submicron sized colloidally deposited Ni-GDC anode functional layer (AFL). The infiltration of Ethanol-based nitrate salts' solution (~2.1 mg cm^−2^) resulted in superimposition of ultra-fine nano-features surrounding a submicron Ni-GDC particulate structure which was retained after high temperature sintering at 1450 °C. The maximum power density of such bi-modally integrated anode reached 1160 mW cm^−2^ (at 600 °C under 30 sccm of 3% wet hydrogen) which was 70% higher than that of the non-infiltrated AFL. The total area specific resistance (ASR) value of the infiltrated sample (0.206 Ω cm^2^) was reduced by 47% in comparison to the non-infiltrated sample (0.387 Ω cm^2^). Sholklapper et al. [27] and Kurokawa et al. [28] reported that infiltration of ceria nanoparticles into composite NiO-based anodes led not only to improved fuel electrode performances but also to a significant increase in its sulphur tolerance. It was shown that at 700 °C the power density of cathode supported cells with traditional Ni-YSZ anode dropped to near 0 mW cm^−2^ in ~13 min when exposed to humidified H_2_ fuel containing 40 ppm H_2_S. The same cells with ceria-infiltrated Ni-YSZ anode delivered power density of ~220–240 mW cm^−2^ for 500 h showing degradation of power density of ~2%. Blennow et al. [29] successfully applied surfactant assisted infiltration of aqueous doped ceria ink plus minor amounts of Ni into ferritic stainless steel/ Y_2_O_3_ Stabilized ZrO_2_ cermet anodes for the production of planar metal-supported SOFCs. The button cells tests demonstrated good long-term stability and excellent performance at temperatures around 650 °C.

This study reports on the feasibility of application of commercially available inkjet printing technology for nano-engineering of SOFC anodes. Inkjet printing was used for high precision infiltration of GDC ink aimed at the optimization of electrochemical activity of composite NiO-GDC anodes. Symmetrical and button cells based on GDC and 8YSZ electrolytes, respectively, and composite NiO-GDC anodes infiltrated with GDC ink were tested for enhanced performances at temperatures at and below 600 °C. Single processing technique was chosen to produce the electrolyte and the anodes as well as to perform the infiltration of the anode, namely drop-on-demand inkjet printing (DoD-IJP). DoD-IJP is a simple and cost effective non-contact “wet” technique applicable on variety of surfaces, which allows utilization of very thin fragile and/or non-even porous supports. It can reproducibly dispense droplets in the range of nL to pL volumes at high rates (kHz). DoD-IJP allows for excellent volume control of jetted drops and hence, precision of the coating thickness and infiltration dosage and distribution. It also introduces the possibility of printing 2D and 3D patterns as well as continuous coatings. Infiltrated anode microstructures were created by consistently depositing nano-liter droplets with micrometre spatial resolution onto porous scaffold surfaces ensuring high reproducibility of the printed procedure.

Inkjet systems offer a wide scale of applications—from experimental platforms working with customized inks, to mass manufacturing systems that can print rapidly and competitively on industrial scale. The technology is cost effective and environmentally friendly through waste minimization of the expensive precursors, which is a critical issue especially for inks based on precious or rare-earth metals. The production of anodes and electrolyte coatings with modified “Domino MacroJet” print head was reported previously by Tomov et al. [30] and Wang et al. [31] using suspension inks. Wang et al. [32] successfully deposited thin GDC electrolytes on NiO-8YSZ cermet anodes using sol–gel-based precursor solutions. Tomov et al. [33] recently demonstrated inkjet printing infiltration of GDC in composite La_0.6_Sr_0.4_Co_0.2_Fe_0.8_O_3−δ_/GDC cathodes.

## Experimental

### Methodology

The infiltration of catalytic or non-catalytic precursor often utilizes ink tailored with suitable surfactants and gelling agents in order to achieve the desired control over the phase and the morphology of the infiltrate nano product—particle size, distribution, wetting angle, etc. Commonly, infiltration procedure is performed in multiple steps. Vacuum treatment could be implemented after each ink delivery step in order to increase the mass load of the infiltrate. An appropriate heat treatment is usually done after each loading step. The infiltrate can form either a nano-decoration composed of discrete nanoparticles or a continuous coating onto the scaffold. In the laboratory environment, such infiltration procedure is usually performed using micro pipettes or sample immersion. The process is cumbersome and slow resulting in non-uniform ink distribution (both lateral and in-depth) and wastage of expensive inks. Few attempts have been reported on scaling up the procedure in order to achieve compatibility with the commonly used ceramic SOFC production routes. Lee et al. [26] transferred the precursor solution to a foam roller which was applied consecutively onto the porous AFL. Kiebach et al. [34] infiltrated stacks of anode-supported SOFCs by “flushing” aqueous solution containing metal nitrates and surfactant through the manifold compartments. While a significant performance enhancement was observed after the infiltration with CGO on the cathode side, the infiltration of the anode side with Ni-CGO had no significant effect on the electrochemical performance. Although providing scalability, both methods have little control over the infiltration process and can lead to substantial non-uniformities and wastage of ink.

**Fig. 1:**
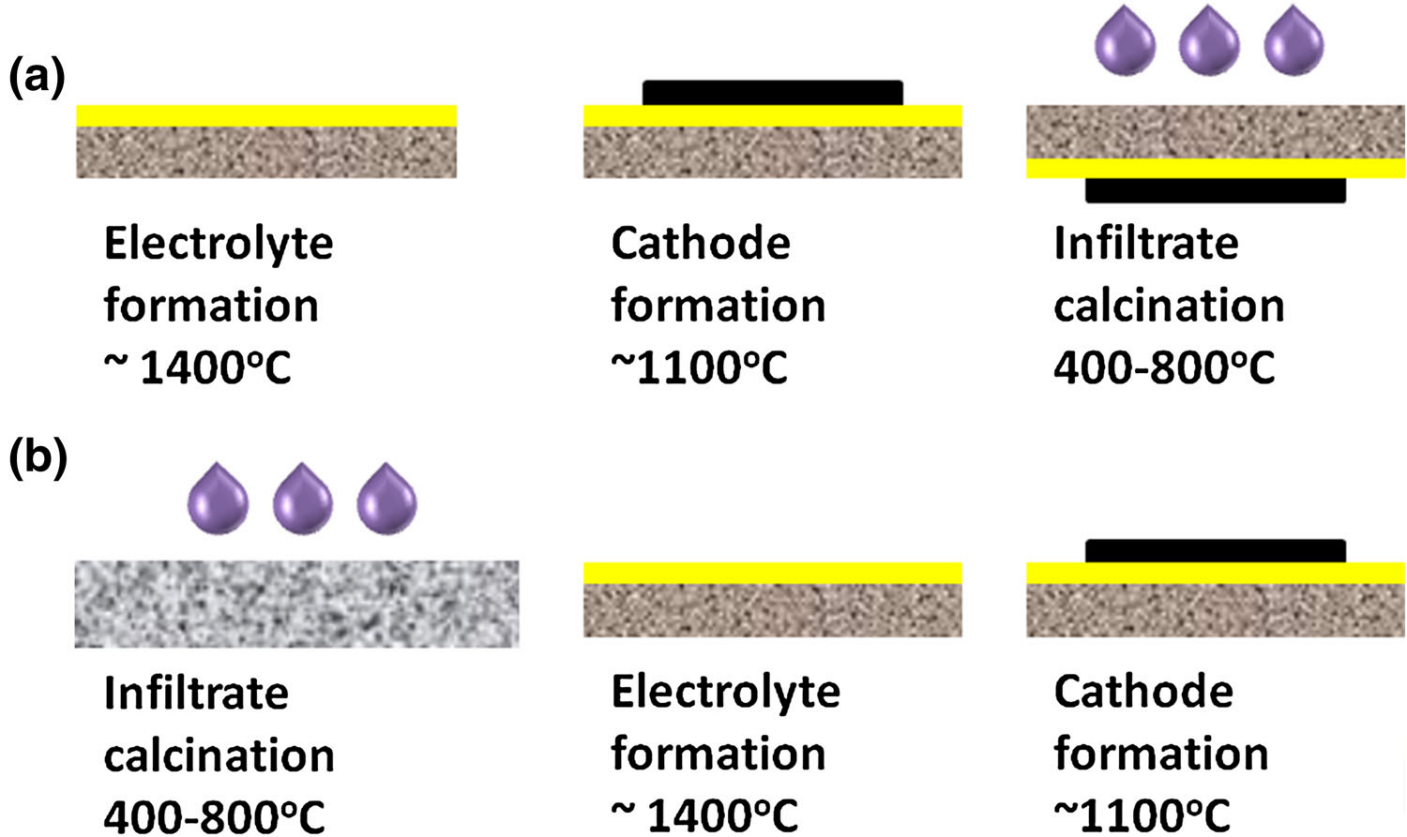
Possible scenarios of anode infiltration along the processing route of SOFC

In terms of the timing, the anode infiltration procedure can be classified into two different scenarios as demonstrated in Fig. 1: (a) infiltration into fully sintered scaffold—mono-phase (e.g. Ni into 8YSZ porous skeleton) or composite (e.g. GDC into Ni-8YSZ composite anode) [34] and (b) infiltration into pre-sintered anode or anode functional layer [26]. The first scenario is performed after the high temperature sintering (~1400 °C) of the electrolyte and the electrode scaffolds (see Fig. 1a). It has the advantage of allowing the infiltrate calcination at low temperatures (~500–800 °C), thus avoiding typical issues such as coarsening of the infiltrated nanoparticles and/or any undesired interaction between the scaffold material and the infiltrate. Although this approach widens the choice of applicable infiltrate materials, an important technological obstacle is the significantly reduced porosity of the anode. This leads to limited loading levels and substantial non-uniformities of the infiltration profiles, especially in the case of thicker anode supports. The second scenario (see Fig. 1b) allows for uniform loading of ink into a pre-sintered anode or AFL prior to the electrolyte formation. Adversely, the high temperature electrolyte sintering step can cause nanoparticles coarsening and undesired chemical interaction between the electrode material and the infiltrate. Thus, the choice of the infiltrate is limited to materials with acceptable inertness and sintering stability.

In this study, we attempted to find a commercially viable non-disruptive technique for SOFC anode infiltration by combining the advantages offered by the second scenario with the precision control of ink dispensation and delivery provided by inkjet printing technology. Our approach implemented inkjet printing infiltration of GDC into the pre-sintered NiO-GDC anode. An optimization of the jetting parameters allowed us to achieve exceptional infiltration uniformity and repeatability with practically no wastage of ink. Variation of the ink rheology and the dynamic of the drops impinging onto the porous surface were used to control the lateral distribution of the ink.

### Inks preparation and jetting optimization

#### Anode and electrolyte inks

10 mol% gadolinium doped cerium (IV) oxide (Sigma-Aldrich), NiO (Sigma-Aldrich) and hydroxypropyl cellulose (Sigma-Aldrich) powders were milled in terpineol with 3YSZ milling balls in a planetary mill for 8 h to ensure average particle sizes of ~400 nm, D90 (measured by Zetasizer 3000HS, Malvern Instruments). Hydroxypropyl cellulose was used as polymeric dispersant for steric ink stabilization (2 wt% of the NiO or CGO powder base). It also acted as a binder and a relief for drying stresses [35] as well as a fugitive agent in the creation of the porous scaffold. The inks were adjusted to the required viscosity range by varying the amounts of binder and diluting solvent. Methanol was chosen as a diluting solvent readily dissolving the polymeric dispersant and allowing for fast drying of the drops. Terpineol (Sigma-Aldrich) was selected as low vapour pressure solvent, inducing Marangoni flows counteracting the formation of coffee-ring stains [36]. It also played the role of a natural dispersant for the oxide particles having excellent miscibility with the polymeric dispersant and the methanol solvent. The dilution at volume ratio 1:1 (terpineol:methanol) was chosen based on stable jetting optimization procedure discussed below.

#### Infiltrate GDC ink

GDC precursor solution (1.5 M total metal concentration) was prepared by dissolving cerium (III) acetate hydrate (Sigma-Aldrich) and gadolinium (III) acetate hydrate (Sigma-Aldrich) in two steps in propionic acid (Sigma-Aldrich) under reflux for 4 h at 120 °C. The solution was then cooled to room temperature and filtered through 0.3 µm polypropylene membrane syringe filter (Whatman) to eliminate possible contamination with dust particles. The precursor was further diluted to reduce the viscosity to a suitable level as determined by the print-head requirements. A number of different solvent/precursor mixtures were tested to evaluate the stability of the ink, which was determined by observing precipitation levels after shelf storage for 24 h. From the various solvents tested 1-propanol was selected as a diluting agent producing the most stable printable inks. The dilution at volume ratio 1:1 (precursor:solvent) was found suitable to produce stable jetting without observing splashing or satellite droplet effects.

#### Jetting optimization

The jetting was performed by electromagnetically (EM) driven single-nozzle print head with a 100 μm orifice, modified from a “Domino MacroJet” printer. Precise control of the drop volume, velocity and satellite drop formation was achieved by optimization of the jetting parameters. Since drop volumes influenced the infiltration resolution as well as the thickness and uniformity of the functional coatings, printing parameters yielding stable jetting of small single drops without satellite drop formation were chosen. The optimisation of the EM print head was based on the variation of the opening time and the nozzle pressure. A custom-built drop visualization system, using a camera (Stingray, Allied Vision Technologies) and a telecentric zoom lens (Moritex) was used to track the drop generation process. Drops were imaged over a distance up to 5 mm corresponding to the experimental distance between the nozzle and the substrates. Drops were back-lit by strobed LED illumination, employing custom-designed electronics to trigger both the strobe and the camera shutter at a selectable delay after the drop ejection.

### Symmetrical cell preparation

GDC powder, hydroxypropyl cellulose and ethanol were mixed and milled for 4 h. Following evaporation of the solvent the powder was uniaxially pressed into pellets with 12.5 mm diameter under 3 tonnes pressure. The pellets were sintered at a heating rate of 3 °C min^−1^ to 900 °C and held for an hour. NiO-GDC (50:50 wt% ratio) suspension inks were printed on both sides of the GDC pellets and fired at 900 °C in air forming porous NiO-GDC anode scaffolds.

An infiltration of the GDC sol ink was carried out, according to the scenario as-described in Fig. 1b, after the formation of the composite anode scaffolds. Sol GDC ink was printed using optimized printing parameters producing ~7 nL drops as determined by drop visualization. Each infiltration step (per side) contained 368 drops (~2.57 μL of ink). A horizontal drop spacing of 0.6 mm was used to achieve an overlap of 25% between the drops surface replicas. Each printed layer was allowed to drain and the absolute position of the print head was offset by 0.3 mm in the X and Y directions to avoid drop stacking. An intermediate heat treatment was applied after every second deposition, increasing the temperature from ~20 to 300 °C within 10 min. Such treatment led to the removal of the solvent from within the scaffold aiding further infiltration steps. Various infiltration loading levels of GDC nanoparticles into the anode scaffold were achieved by varying the number of printing steps. Finally, as treated pellets were sintered at 1400 °C for 4 h in order to achieve fully dense electrolytes. As sintered symmetrical cell were ~0. 5 mm thick and ~11 mm in diameter.

### Button cells preparation

GDC anode infiltrated button cells were produced following the scenario as-described in Fig. 1b. Commercial (CEREL-Poland) anode substrates (50 wt% NiO, 50 wt% 8YSZ) prepared by tape casting and pre-sintering at 1100 °C were used as supports. NiO-GDC (50:50 wt% ratio) functional layer was inkjet printed on one side on the anode support and fired at 900 °C in air forming porous NiO-GDC anode scaffold. GDC sol ink was infiltrated into as-sintered porous scaffold copying the procedure used for the preparation of the symmetrical cells (described in Sect. 2.3). In the next step, a GDC electrolyte was inkjet printed onto the infiltrated surface using GDC suspension ink. The anode/electrolyte assembly was subsequently sintered at 1400 °C for 4 h to obtain a fully dense electrolyte membrane. As sintered cells were ~1.0 mm thick and ~20 mm in diameter. Commercially available cathode La_0.6_Sr_0.4_Co_0.2_Fe_0.8_O_3−*δ*_ (LSCF) powder (Nextech Inc.) was dispersed with GDC powder (50:50 wt% ratio) in a mixture of alpha-terpineol and ethanol plus dibutyl phthalate (DBP) and polyvinyl butyral (PVB) (plasticiser and binder, respectively). The ink was screen printed onto the GDC electrolyte forming circular cathode electrode ~14 mm in diameter. An additional current collector (LSCF paste, Nextech) layer with the same size was also screen printed on top of the cathode layer. The samples were subsequently sintered at 1200 °C for 2 h. For comparison, a reference cell was produced following the same procedure as above but without GDC sol ink infiltration.

### Characterization

The electrochemical performance of the symmetrical Ni-GDC/GDC/Ni-GDC cells was characterized by 2-point electrochemical impedance spectroscopy (EIS) technique. EIS was performed with a Solartron 1260 impedance/gain phase analyzer in conjunction with a Solartron 1287 electrochemical interface. The frequency range from 0.1 Hz to 1 MHz was used with amplitude of 10 mV. The electrode performance of the reference and the impregnated anodes was measured in 500–600 °C range at 50 °C intervals in humidified 4% hydrogen/argon mixture with a gas flow rate of 30 mL min^−1^. The anodes were reduced in-situ at 800 °C for 1 h prior the test. The microstructure of the scaffolds was examined by FEGSEM (JEOL 6420). Button fuel cell (Ni-8YSZ/Ni-GDC/GDC/LSCF-GDC) were sealed to zirconia tube with a glass seal. Pt paste meshes were painted onto both electrodes creating contact areas of ~ 0.78 mm^2^. Pt wires were attached to the mesh for current collection. Hydrogen gas humidified at room temperature (∼3% H_2_O) was used as fuel. Flow rate of 60 mL min^−1^ of humidified H_2_ was supplied to the anode side while the cathode was open to ambient air. The open circuit voltage (OCV) and current–voltage (*i*–*V*) behaviour were measured at 600 °C.

## Results and discussions

**Fig. 2:**
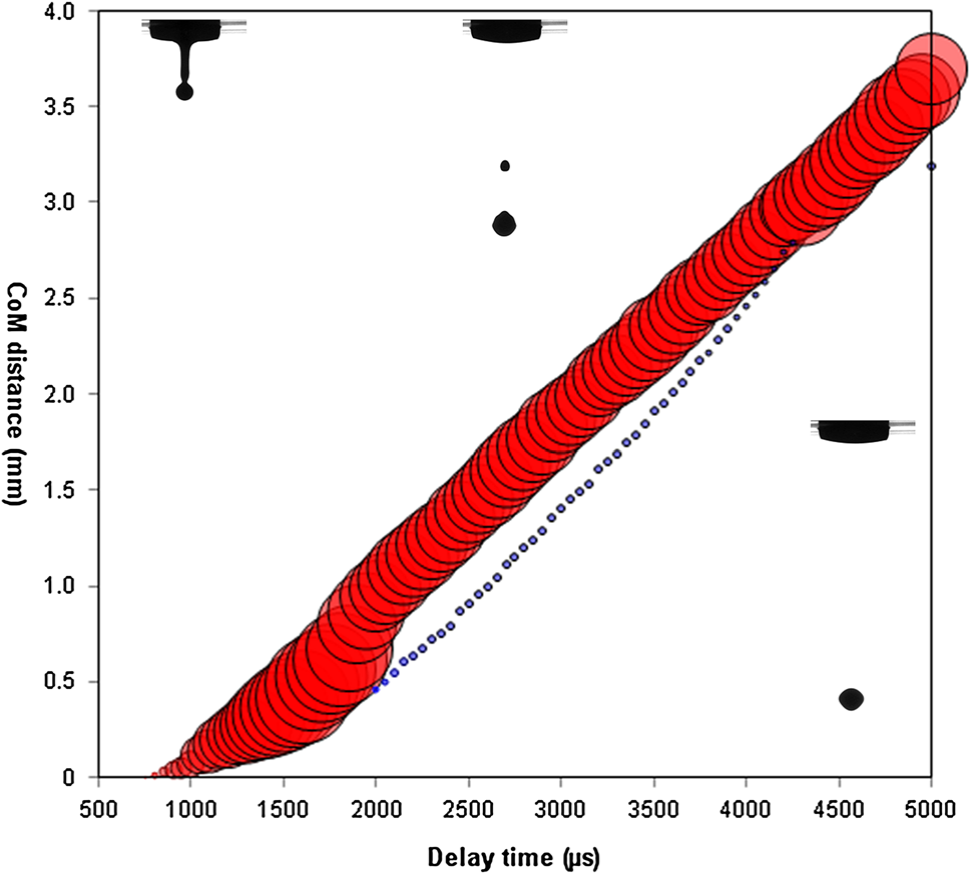
Variation of the centre of mass (CoM) of the drops with the delay time generated by the drop visualization system and showing jetting of the GDC sol ink under optimized printing parameters

The drop formation behaviour of GDC sol ink is illustrated in Fig. 2 presenting the variation of the centre of mass (CoM) of the drops with the delay time. In the graph, automatically generated by the proprietary drop imaging software, colour red represents the main drop and colour blue the secondary drops. The diameters of the circles are proportional to the actual size of the drop calculated at different delay times. It can be observed that for the chosen optimized jetting parameters (pressure of 200 mbar and an opening time of 300 µs), an initial drop broke into two drops approximately 2000 μs after the triggering event. The smaller satellite drop caught up with the main drop after ~4000 μs and formed back to a single drop of ~7 nL volume. Images of drop (drops) in flight are superimposed on the graph illustrating drop behaviour observed at different delay times. Having no satellite drops impinging onto the surface of the substrate, one can insure that the delivery of ink over the whole surface area is highly uniform and controllable within the limits of the instrumental resolution of the X–Y table. The same procedure was performed for the NiO-GDC suspension ink. Within the stable jetting regime, it was found that the combination of 330 mbar and 250 µs yields the smallest drop volume of ~22 nL. The fluctuation in the estimated drop volumes (less than 5% of the total volume) was caused by the uncertainty arising from image thresholding, image resolution and the volume calculation procedure which assumed ideal rotationally symmetrical drops.

**Fig. 3:**
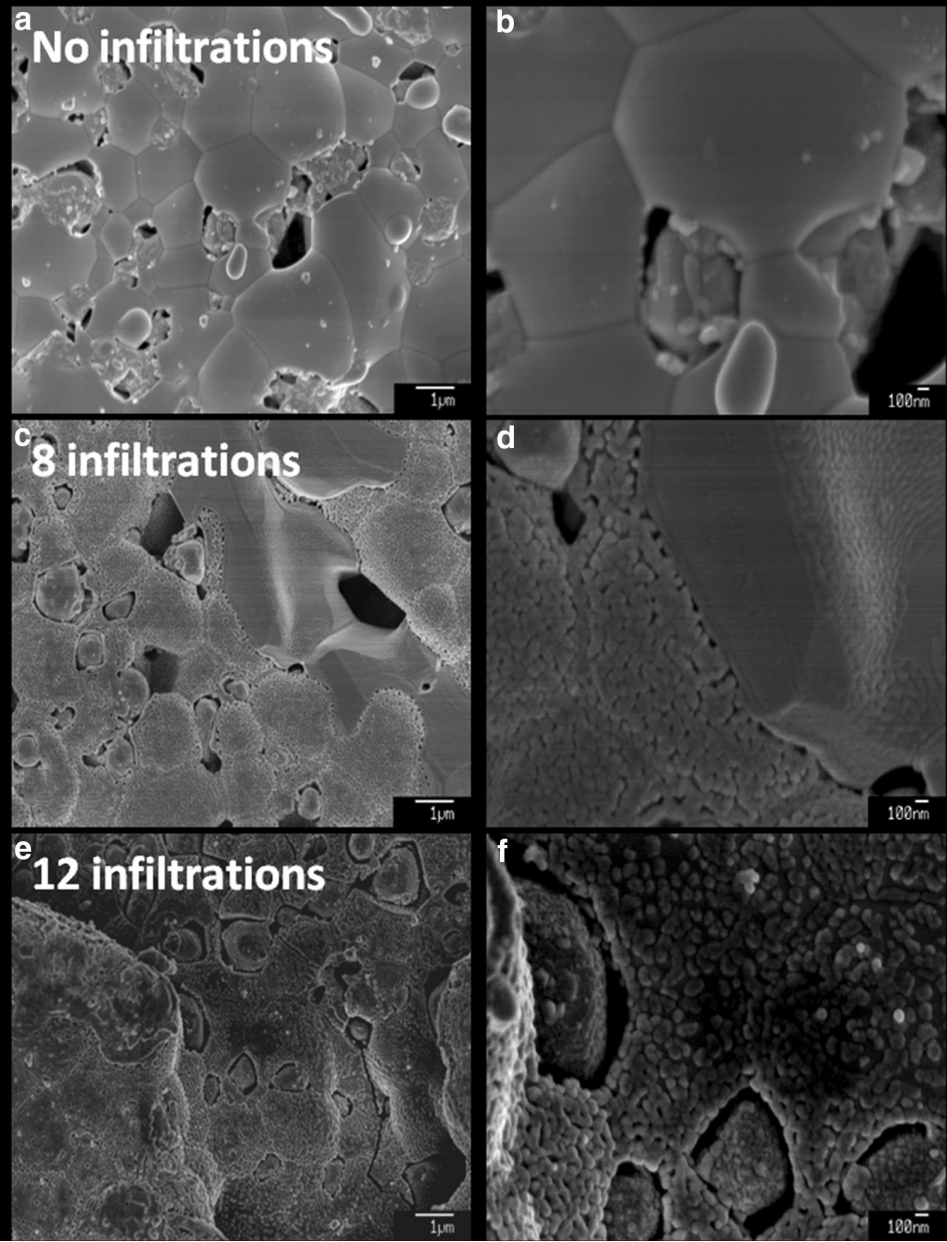
SEM images of: **a**, **b** anode scaffold without infiltration, **c**, **d** anode scaffold with 8 infiltration/heating cycles and **e**, **f** anode scaffold with 12 infiltration/heating cycles

Multiple infiltration cycles were conducted by repeated inkjet printing/low temperature heating (0, 8, 12 and 20 infiltration steps) in an attempt to create percolating GDC nano-decorations. It is worth noting that 20 cycles were found to be the empirical infiltration limit for the in-house produced pre-sintered NiO-GDC composite anodes without application of high temperature calcination steps. As seen in the high resolution SEM images shown in Fig. 3, the NiO-GDC scaffolds were composed of well connected NiO and GDC grains with sizes ranging between 1 and 3 μm (Fig. 3a, b). After the infiltration, the scaffolds were uniformly decorated with GDC nanoparticles forming nano-scale sub-structures. Partial coverage with GDC nanoparticles was seen after 8 infiltration cycles (Fig. 3c, d), whereas complete coverage of the scaffold was achieved at 12 infiltration cycles (Fig. 3e, f). Further infiltration (20 cycles) did not lead to distinguishable changes of the scaffold surface morphology. All infiltrated samples showed similar sub-structures with GDC nanoparticle size varying in the range of 50–200 nm. Thus the infiltrated nano sub-structure was retained after the high temperature sintering at 1400 °C. Similar observation was reported by Lee et al. [26] for Ni-GDC infiltration in AFL. The exact nature of this effect is not clear as according to Rupp et al. [37] self-limited grain growth for nano GDC characterized by grain boundary diffusion was observed for temperatures below 1100 °C. One could speculate that the retention of the nano sub-structure could be related to the strong bonding between the GDC nanoparticles and the micro-strain defects in the anode scaffold.

**Fig. 4:**
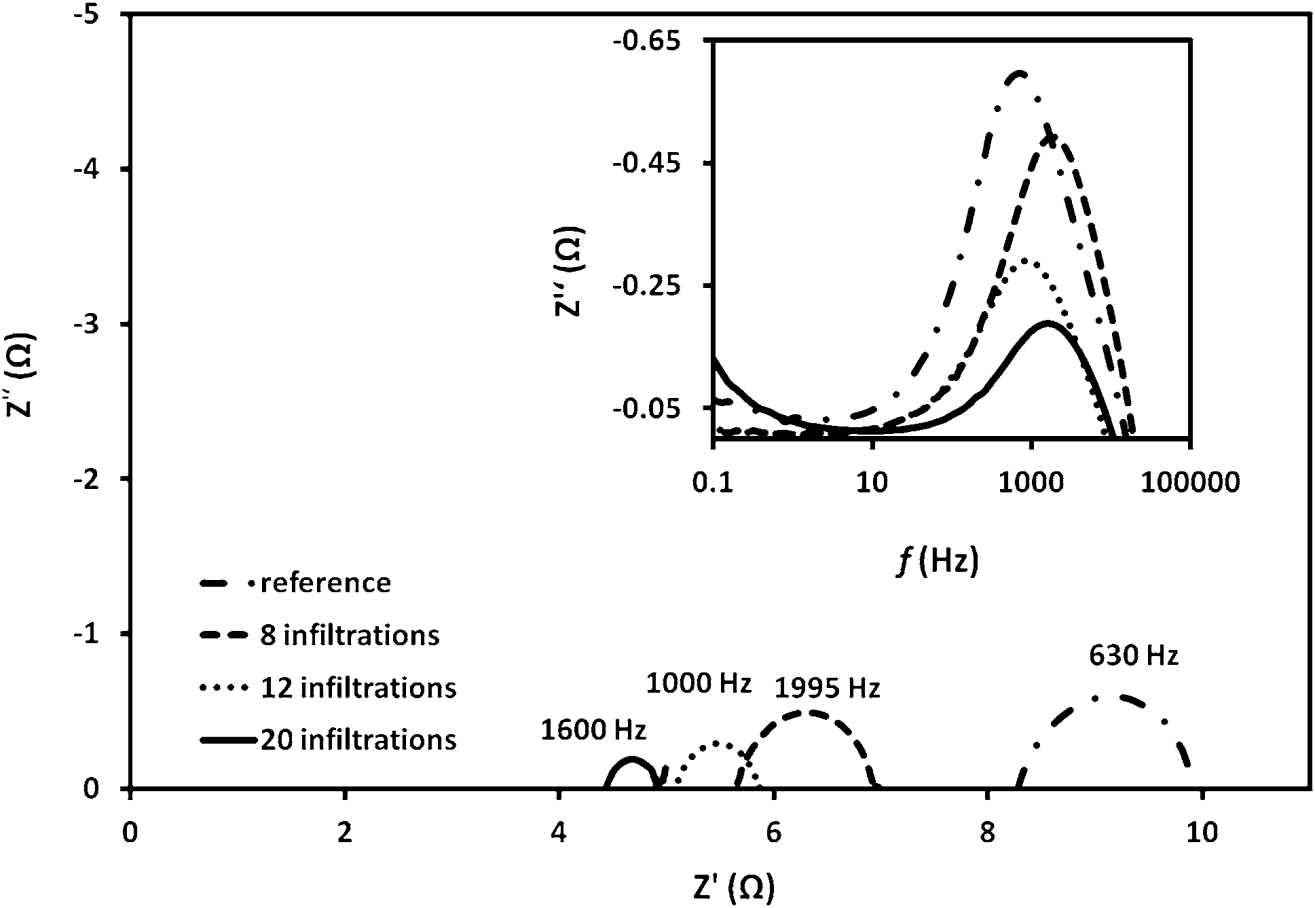
EIS spectra of anodes with 0 (reference), 8, 12 and 20 infiltrations at 550 °C. The inset shows the frequency dependence of the impedance imaginary part for different number of infiltrations

A quantitative confirmation of the effect of the number of infiltration cycles on the electrochemical performance was pursued by EIS testing of as-prepared symmetrical cells. Comparison of the Nyquist plots of the reference anode and anodes with various number of infiltration cycles (8, 12, 20) at 550 °C is shown in Fig. 4. The summit frequencies are shown above each semi-circle. The EIS spectra for all measured symmetrical cells were similar in shape, showing single arc associated with the activation polarization resistance (*R*_p_) at the anode without clear separation between low and high frequency arcs. After GDC impregnation, the overall impedance arcs were systematically reduced with the increasing number of infiltration cycles. The total (*R*_t_) and ohmic (*R*_s_) polarization resistances were estimated from the low and high frequency intercepts of the Nyquist plots with the real axis. *R*_p_ was calculated from the difference between these two values divided by two—*R*_p_ = (*R*_t_ – *R*_s_)/2 in order to account for the symmetrical nature of the cells. All polarization resistances henceforth are given for one electrode. The area specific resistance (ASR) was obtained from the polarization resistance by multiplying *R*_p_ with the anode area. After the infiltration and formation of GDC nanodecorations a significant reduction of ASR values was registered. The ASR of the reference (non-infiltrated) anode was measured as 2.95, 0.81 and 0.41 Ω cm^2^ at 500, 550 and 600 °C, respectively. The ASR of the NiO-GDC composite anode with 12 infiltration/heating cycles was 1.11, 0.37 and 0.15 Ω cm^2^ at 500, 550 and 600 °C, respectively.

A direct comparison of the results reported by various groups on infiltration of SOFC anodes with doped ceria is difficult due to the variation of reported figures of merit and the lack of convention on the measure of the infiltrate loading. A number of parameters were reported having influence on the outcome of the infiltration procedure and they alter significantly between different studies—e.g. type of the scaffold (mono-phase or composite), type of the cell (symmetrical, button), thickness of the anode, type of the ink (solvent and additives), number of the infiltration steps and use of vacuum to assist the ink penetration between steps, calcinations temperatures (*T*_calc_) and durations, testing EIS temperatures (T_EIS_). In order to evaluate the results of the present work we have summarized the data reported by several groups on similar infiltrations of anodes with doped ceria inks (see Table 1). The promotion factor—*ε*_ASR_ = ASR_ref_/ASR_inf_—was chosen as a figure of merit where ASR_ref_ were the ASR values of the reference (non-infiltrated) anode and ASR_inf_ were the ASR values of the infiltrated anodes. As seen in the Table 1 the value of ***ε*** varies with the temperature and the type of infiltration procedure between 1.33 and 3.45. An exception of this trend is the result reported by Jiang et al. [20, 21] on the infiltration of thin pure NiO anodes. The values of ***ε*** measured in this work varied with the temperature at levels comparable and exceeding those reported in the literature—(−*ε*_ASR_ (500 °C) = 2.6; −*ε*_ASR_ (550 °C) = 2.2; −*ε*_ASR_ (600 °C) = 2.8). Those were achieved with almost with lower ink expenditure in a single step infiltration procedure without intermediate high temperature calcinations.

**Table 1:** Improvement factor (*ε*) of NiO-based anodes infiltrated with doped ceria

Source	Infiltrated ink	Scaffold/cell type	Solvent and additives	*T*_calc_ (°C)	*N*_inf_	*T*_EIS_ (°C)	ASR_ref_ (Ω cm^2^)	ASR_inf_ (Ω cm^2^)	*ε*	Loading levels (method of loading)
Jiang et al. [20, 21]	Gd_0.1_Ce_0.9_O_2−*x*_ 3.0 M mol L^−1^	NiO S-FC	Not specified	850	2	700	10.70	1.50	7.13	1.7 mg cm^−2^; 8.5 vol.% (dropper)
						900	1.90	0.22	8.63	
Jiang et al. [22]	Nano-YSZ suspension	NiO S-FC	Not specified	850	2	700	10.7	3.10	3.45	4.0 mg cm^−2^; 21 vol.% (dropper)
						800	3.30	1.50	2.20	
						900	1.90	0.50	3.80	
Jiang et al. [23]	Sm_0.2_Ce_0.8_O_1.95_ 0.25 M mol L^−1^	NiO/TZ3Y ES-FC	Not specified	1000	1	800	1.74	1.1	1.58	0.5–2.4 mg cm^−2^ (dropper, vacuum)
Liu et al. [24]	Sm_0.2_Ce_0.8_O_1.95_ 1.0 M mol L^−1^	NiO/Sm_0.2_Ce_0.8_O_1.95_ AS-FC	Distilled water	800	4	600	0.288	0.216	1.33	1.71 mmol cm^−3^ (dropper, vacuum)
Lee et al. [26]	NiO-Gd_0.1_Ce_0.9_O_2−*x*_ 1.0 M mol L^−1^	NiO/Gd_0.1_Ce_0.9_O_2−*x*_ AS-FC	Ethyl alcohol	1450	1	600	0.285	0.136	2.09	2.1 mg cm^−2^ 2.3 wt% (foam roller, 80 °C)
This work	Gd_0.1_Ce_0.9_O_2−*x*_ 0.75 M mol L^−1^	NiO/Gd_0.1_Ce_0.9_O_2−*x*_ S-FC	Propionic acid—C_3_H_6_O_2_	1400	1	500	2.95	1.11	2.6	30 μL ink per electrode; ~5 mg cm^−2^ (12 infiltration printing steps)
						550	0.81	0.37	2.2	
						600	0.41	0.15	2.8	

*N*_inf_ number of infiltrations/calcinations cycles, *S*-*FC* symmetrical fuel cell, *ES*-*FC* electrolyte supported fuel cell, *AS*-*FC* anode-supported fuel cell; *T*_calc_ calcinations temperature; *T*_EIS_ testing temperature

**Fig. 5:**
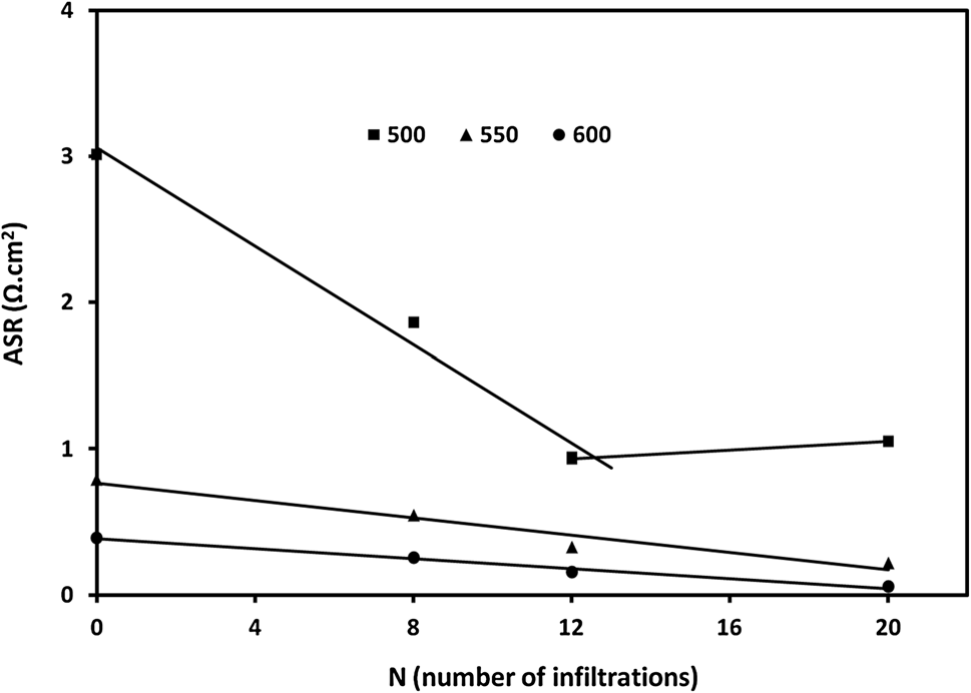
Variation of the electrodes’ ASR values with the number of infiltration/heating cycles (*N*) at different testing temperatures (500, 550, 600 °C) (note that the *straight lines* are linear fit of the experimental data)

**Fig. 6:**
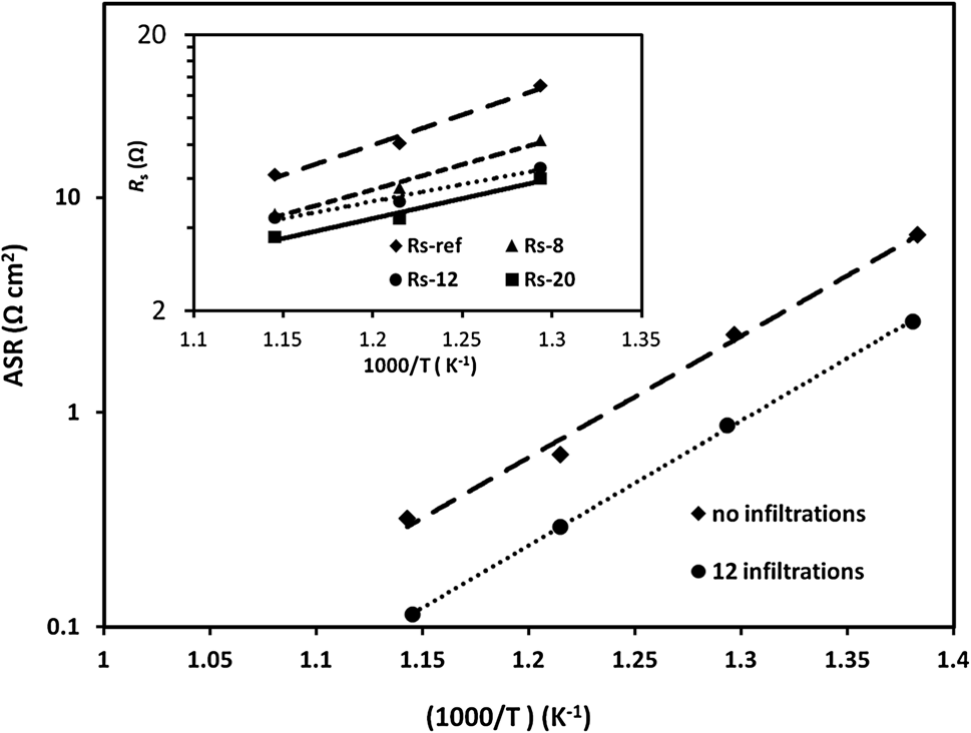
ASR values versus temperature plots of NiO-GDC anodes without and with 12 infiltration/heating cycles. The inset shows the variation of the ohmic resistance (*R*_s_) with the temperature for different number of infiltrations (note that the straight lines are linear fit of the experimental data)

**Fig. 7:**
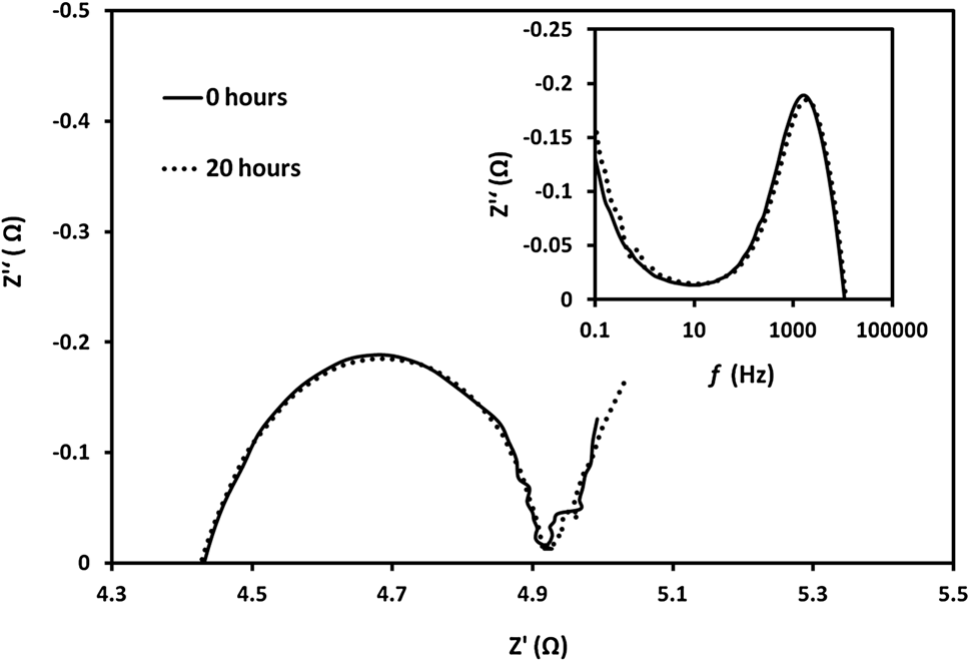
The impedance spectra of anode with 20 infiltration/heating cycles aged at 600 °C and tested at 550 °C. The inset shows the frequency dependence of the impedance imaginary part

Summary of the dependence of ASR_inf_ versus the number of infiltrations at different testing temperatures is presented in Fig. 5. It was apparent that ASR_inf_ was linearly dependent on the number of infiltrations which we assign to the proportional extension of TPB. Exception from this trend was the ASR_inf_ value found al the lowest testing temperature (500 °C) and the highest number of infiltrations—20. This was related to the expected localised agglomeration of GDC nanoparticles at high loading levels effectively reducing the length of the TPB. The frequency dependencies of the imaginary impedance components (*Z*″) for 12 infiltration cycles are presented in the inset graph of Fig. 4. The summit frequency of the reference sample and the infiltrated samples varied between 630 Hz and 1600 Hz. Figure 6 shows the activation energy plots of the polarization resistance of NiO-GDC anodes without infiltration and with 12 infiltration/heating cycles. The activation energies (*E*_a_) were estimated from the slope of the linear of the experimental data—*E*_a_ = 108 kJ mol^−1^ for the reference anode and *E*_a_ = 111 kJ mol^−1^ for the infiltrated anode. These activation energy values were typical for the Ni-CGO/CGO/Ni-CGO symmetrical cell reported in the literature—*E*_a_ = 140 kJ mol^−1^ was reported by Almar et al. [38] for mesoporous Ni-CGO anodes; Muecke et al. [39] found that thin film Ni-GDC anodes exhibited *E*_a_ = 145 kJ mol^−1^; Galinski et al. [40] reported *E*_a_ = 164 kJ mol^−1^ for sprayed Ni-40GDC anodes. The relatively high summit frequencies, the similarity of the activation energies over the measured temperature range and the linearity of ASR_inf_ versus N dependence suggested that a charge transfer process was the most likely rate limiting step among the reactions occurring in the infiltrated anode [16, 23, 30]. Hence, the impregnation of mixed ionic and electronic conductive oxide (GDC) into a composite NiO-GDC scaffold led to the extension of TPB by percolating GDC nano-decoration. The inset in Fig. 6 shows the activation energy plots for the Ohmic resistances (*R*_s_) for all measured anodes. The consistent drop in *R*_s_ with the number of infiltrations could be assigned to the extension of the 2D contact area between the electrolyte and the anode and possible “healing” of micro-cracking defects at the scaffold grain boundaries. A development of concentration polarization arc was clearly detectable at low frequency end of the Nyquist plots for the cell with highest number of infiltrations (20 infiltration/heating cycles) which was evidence for a porosity reduction of the scaffold by the infiltrate. Finally, the sample with 20 infiltration cycles was subjected to temperature degradation (ageing) test at 600 °C for 20 h. As shown in Fig. 7, very small changes in the polarization resistances were observed during testing for 20 h. Additionally, summit frequencies (*f*_summit_) as seen in the inset of Fig. 7 were found to be consistently stable thus reflecting the durable performance of as infiltrated anode.

**Fig. 8:**
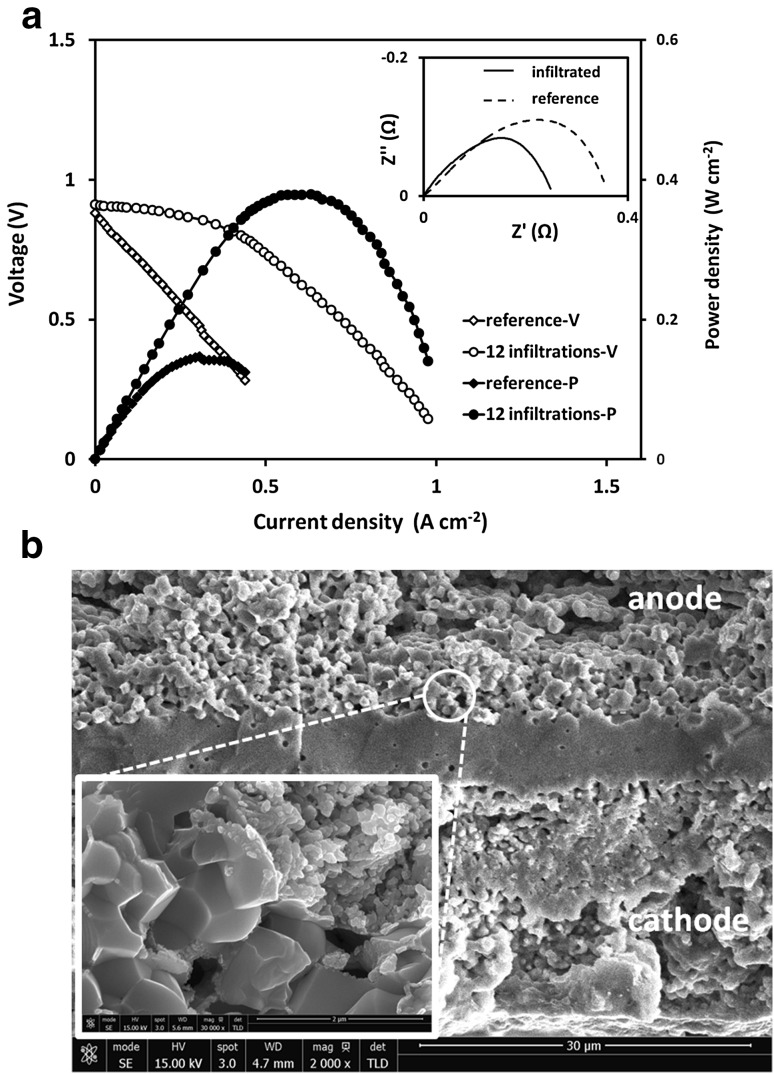
**a** The effect GDC nano-decoration on the cell *i*–*V* characteristics using 60 mL min^−1^ of humidified hydrogen and ambient air at 600 °C. The circles represent power density data and diamonds represent potential data. The inset shows the Nyquist plots of both button cells; **b** cross-sectional SEM image of the infiltrated (12 infiltrations) button cell

Figure 8a presents the *i*–*V* characteristics of both, the GDC infiltrated (12 infiltrations) and the reference button cells, measured at temperature of 600 °C in humidified hydrogen (gas flow rate of 60 mL min^−1^). The inset in Fig. 8a demonstrates the effect of GDC infiltration on the Nyquist plots of the button cells which included polarization resistance contributions from both anode and cathode. As both cells had identical cathodes the reduction of the ASR value was assigned to the effect of nano structuring of the active anode area with infiltrated GDC nanoparticles. A promotion factor *ε*_ASR_ = 1.5 was estimated from the polynomial fit of the spectra. As expected this factor was lower than the one estimated for the symmetrical cells with 12 infiltrations (*ε*_ASR_ = 2.8) due to the constant sizable contribution of the cathode polarization resistance. Note that the plots were shifted to subtract the ohmic component for better comparison. Figure 8b shows cross-sectional SEM image of the infiltrated cell. Higher magnification of the anode-electrolyte area shown in the inset of Fig. 8b reveals the infiltrated GDC nanostructure created by the inkjet printed infiltration. The cells exhibited an open circuit voltage (OCV) of 0.85 V for the reference cell and 0.90 V for the infiltrated cell, which is typical for SOFCs with GDC electrolytes at 600 °C. The infiltrated cell exhibited maximum power density (MPD) of 380 mW cm^−2^ which assumed MPD promotion factor *ε*_MPD_ = 2.53 (where *ε*_MPD_ = MPD_inf_/MPD_ref_; MPD_inf_ is maximum power density of the infiltrated sample and MPD_ref_ is maximum power density of the reference sample). Maximum power density of 150 mW cm^−2^ was measured for the non-infiltrated cell. The MPD for the infiltrated sample was obtained at current density of ~0.6 A cm^−2^ but suppressed by concentration polarization starting at 0.4 A cm^−2^. This effect was related to gas diffusion limitations through the anode introduced by the infiltration and the non-optimized cathode microstructure as seen in Fig. 8b. Table 2 makes comparison between values for the open circuit potential (OCP), ASR, MPD and *ε*_MPD_ measured in this work and the results published by Lee et al. [26]. The cells infiltrated by Lee et al. [26] had similar architecture and materials selection but were infiltrated with mixed Ni-GDC precursor using foam roller (reaching loading level of 2.1 mg cm^−2^). While having similar OCP, ASR and *ε*_MPD_ confirming the efficiency of inkjet printing infiltration procedure, the MPD measured in this work was significantly lower. This was ascribed to the non-optimized cathode deposition and sintering procedures.

**Table 2:** Comparison of OCV, ASR, MPD and *ε*_MPD_ values measured in this work and data reported by Lee et al. [26]

	OCP (V)	ASR (Ω cm^2^)	MPD (mW cm^−2^)	*ε*_MPD_
Lee et al. [26]	0.85	0.190	1156	1.68
This work	0.09	0.136	380	2.53

## Conclusions

It was demonstrated that the anode microstructure could be successfully nano-engineered by inkjet printing infiltration. DoD Inkjet printing enabled high precision infiltration of a GDC sol ink allowing formation of GDC nano-decoration on the existing composite anode scaffold. The modified anode scaffolds showed significant enhancement of their electrochemical performance with the improvement factor for 12 infiltration/heating cycles in the range of *ε* = 2.2–2.8 which was comparable with the previously reported results on vacuum assisted infiltration of doped ceria into composite anodes. Similarly, treated button cells produced an increase of the maximum output power of the similar order (~2.5 times). The correlation between EIS and *i*–*V* tests suggested proportionality between the enhancement of the TPB and the presence of faster reaction rates at the nano-decorated anode. Considering the non-disruptive character of the method, the minimal expenditure of the ink, the maturity of the inkjet printing technology and the potential for scale-up, inkjet printing infiltration was shown to be a feasible solution for the commercialization of the infiltration technique in SOFC technology.
